# Prospects of Immunotherapy for Triple-Negative Breast Cancer

**DOI:** 10.3389/fonc.2021.797092

**Published:** 2022-01-17

**Authors:** Dan Qiu, Guijuan Zhang, Xianxin Yan, Xinqin Xiao, Xinyi Ma, Shujun Lin, Jieyan Wu, Xinyuan Li, Wandi Wang, Junchen Liu, Yi Ma, Min Ma

**Affiliations:** ^1^ School of Traditional Chinese Medicine of Jinan University, Jinan University, Guangzhou, China; ^2^ School of Nursing of Jinan University, Jinan University, Guangzhou, China; ^3^ School Public Health, Southern Medical University (No: 3210090112), Guangzhou, China; ^4^ School of Medicine, Jinan University, Guangzhou, China; ^5^ Department of Cellular Biology, Institute of Biomedicine, National Engineering, Research Center of Genetic Medicine, Key Laboratory of Bioengineering Medicine of Guangdong Province, The National Demonstration Center for Experimental Education of Life Science and Technology, Jinan University, Guangzhou, China; ^6^ The First Affiliated Hospital of Jinan University, Jinan University, Guangzhou, China

**Keywords:** triple-negative breast cancers, immunotherapy, immune checkpoint molecules, PD1/PD-L1 pathway, CTLA-4, combination therapy

## Abstract

In the classification and typing of breast cancer, triple-negative breast cancer (TNBC) is one type of refractory breast cancer, while chemotherapy stays in the traditional treatment methods. However, the impact of chemotherapy is short-lived and may lead to recurrence due to incomplete killing of tumor cells. The occurrence, development, and relapse of breast cancer are relevant to T cell dysfunction, multiplied expression of related immune checkpoint molecules (ICIs) such as programmed death receptor 1 (PD-1), programmed cell death 1 ligand 1 (PD-L1), and cytotoxic T-lymphocyte-associated antigen 4 (CTLA-4) produce immunosuppressive effect. Immunotherapy (namely, immune checkpoint inhibitors, adoptive cellular immunotherapy, CAR-T immunotherapy and some potential treatments) provides new hope in TNBC. This review focuses on the new immune strategies of TNBC patients.

## Introduction: Treatment and Prognosis of TNBCs

According to the statistics of the World Health Organization (WHO), approximately 8.2 million people beings die of most cancers every year, accounting for 13% of international deaths. As one of the oldest tumors in the records of human civilization, breast cancer is the most clinically diagnosed cancer (1). In the classification and classification of breast cancer, breast cancer that does not express estrogen receptor (ER) or the progesterone receptor (PR) and does not amplify ERBB2 [commonly called human epidermal growth factor receptor 2 (HER2)] amplification are categorized as triple-negative breast cancer (TNBC), accounting for 10–20% of all breast cancers. TNBC subtypes were categorized by multi-omics data (2): (1) Intracavity androgen receptor subtype characterized by means of androgen receptor signal (23%); (2) Immunomodulatory (IM) subtype (accounting for 24% of tumors) with excessive immune cell signal and cytokine signal gene expression; (3) A basal-like and immune-suppressed (BLIS) (39%) subtype, characterized with the aid of upregulation of cell cycle, activation of DNA restore and downregulation of immune response genes; and (4) a mesenchymal-like (MES) subtype rich in breast stem cell pathway (15%). In addition, in the clinical patient population, we can see that TNBC is more common in young female patients. The tumor is usually large in size and of high grade, with greater lymph node metastasis at diagnosis, and has a high biological aggressiveness. Compared with women with other breast cancer subtypes, female with TNBC have higher early distant recurrence rate and worse 5-year prognosis. Therefore, it is very indispensable to obtain the cure purpose at an early stage or manipulate disorder inside the controllable range. Currently, the essential scientific remedies for TNBC consist of surgical resection, chemotherapy, radiotherapy, targeted therapy (3). Conventional chemotherapy drugs, including paclitaxel, anthracycline and alkylating agents, are prone to systemic toxicity and side effects. In addition, as patients with advanced TNBC are highly metastatic and aggressive, it is difficult to achieve good results with targeted therapy or hormone therapy alone (4).

## TNBCs and Immunotherapy

In general, the immune system of healthy individuals is strong enough to shortly get rid of the mutated most cancers cells, while the immune function of most cancer patients can’t successfully recognize and kill tumor cells (5). On the other hand, most tumor cells have many distinct mechanisms to defend them from being identified by means of immune cells (6). Different from the traditional therapies mentioned above, immunotherapy cannot efficaciously kill most tumor cells alone, however additionally decorate the immunity of patients, in particular in the removal of minimal residual lesions and drug-resistant tumor cells. It can keep away from many shortcomings of other therapies to the greatest extent (7). Cell immunotherapy, as a new technology with targeted killing effect on tumor cells, has achieved good results in clinical application in recent years.

### Immune Checkpoint Inhibitors

ICIs are inhibitory molecules expressed on the cell surface, which are usually involved in regulating the activation of T cells. Basically, its most essential feature is comparable to the braking device of an auto-cell, which makes it “brake” in time when the immune system is activated, continues the activation of the immune system within normal limits, and avoids over-activation of the immune system. No matter whether overexpression or over-function of immune checkpoint molecules leads to suppression of immune function, resulting in low immunity and susceptibility to tumor and other diseases (8). Another way to think about it is that if the immunosuppressive function of checkpoint molecules is poor, the immune function of the body will be abnormal. Recent studies have shown that molecular pathways of immune checkpoints, such as programmed death ligand 1 (PD-L1) and programmed death ligand 2 (PD-L2), play a very important negative regulatory role in tumor immunity (9–11).

#### CTLA-4 and TNBC

It is conventional that CTLA-4 is a negative regulator, which is very vital for T cell-mediated immunity. In T cells, CTLA-4 and CD28 bind to the equal ligands (CD80 and CD86) on antigen imparting cells and have contrary effects. The interplay between CTLA-4 and its ligand inhibits T cell reaction, and when CD28 and its ligand bind, T cell reaction is activated. The affinity of CTLA-4 to CD80/CD86 is greater than that of CD28 (12). The upregulation of CTLA-4 in cancer patients is considered as an important mediator of immune escape. Studies have shown that tumor cells of TNBC patients express CTLA-4 in different cell compartments (13). Its foremost ligand, CD80/CD86, is expressed in TNBC cell lines and tumors. This means that blocking CTLA-4 with Ipilimumab (anti-CTLA-4 monoclonal antibody, which has been accredited as checkpoint inhibitor for melanoma treatment) can significantly activate the molecular cascade, which may help enhance the immune response to tumor cells (14). CTLA-4 expressed on the surface of tumor cells during the treatment of patients with TNBC may be the target of checkpoint inhibitors and a candidate biomarker for immunotherapy. In a word, we believe that the operation or chemotherapy of TNBC patients, not only can the combination of targeted immune checkpoint drug therapy play a synergistic role to a great extent, but also can increase the cure probability of cancer patients.

#### PD-1 and PD-L1

PD-1 antibody is a most researched and clinically developed immunotherapy. PD-1 is expressed in activated T cells, B cells, and myeloid cells. It has two ligands, PD-L1 and PD-L2. The binding of PD-1 and PD-L1 mediates the co-inhibitory signal of T cell activation, suppresses the killing function of T cells, and performs a negative regulatory role in human immune response (15–17). In a normal immune system, PD-1 is up to preserve the position of immune tolerance. Tumor cells can escape immune surveillance through immune escape. Targeted therapy based on immunosuppressive receptors and immunosuppressive checkpoint immunotherapy based on immune molecules are new hotspots in oncology research (18, 19). It is additionally discovered that PD-L1 binds to PD-1 receptor on activated T cells and weakens anti-tumor immunity by inhibiting T cell activation signal. PD-1^+^ T cells can partially recover by blocking PD-1/PD-L1 signaling pathway (20–24). Some studies have proven that PD-L1 antibody combined with paclitaxel is effective in treating advanced in the treatment of advanced TNBCs (25). TNBC subtype research based on multi-group data shows that immunoglobulin subtype has high immune cell signal (2). Both clinical and economic characteristics indicate that immune recognition is activated in IM subtype, which shows that the mechanism of immune break out of these tumors may additionally contain the recruitment of immunosuppressive cells or the activation of immune checkpoint molecules. Based on what has been discussed above, we may conclude that high expression levels of immune checkpoint suppressor genes such as PD1, PD-L1, cytotoxic T-lymphocyte-associated antigen 4 (CTLA4), and IDO1 (Indoleamine 2,3-dioxygenase 1) may inhibit the activation of the immune system and lead to the occurrence of TNBC. A Phase III trials confirmed that Atezizumab (PD-L1 inhibitor) and nabo-paclitaxel in the treatment of advanced TNBC, compared with placebo + nabo-paclitaxel, atezizumab + nabo- Paclitaxel can significantly improve the progression-free survival (PFS) (7.5 months vs 5.5 months, respectively) and overall survival (OS) (25.0 months vs 15.5 months, respectively) (26). The response rate of TNBC to ICIs is higher than that of hormone-receptor positive and HER2-positive breast cancers. Recently, the inhibitory effect of carbamazepine plus apatinib on PD-1 in advanced patients in Phase II trial was found, and carbamazepine plus apatinib had good tolerance and showed good ORR (43.3%) and PFS in advanced patients, regardless of lines of therapy and PD-L1 status [NCT03394287] (27). Furthermore, PD-1/PD-L1 inhibitors combined with chemotherapy are more successful in TNBC than single dose ICIs. These results indicate that combinations with chemotherapy could increase the response rate to immunotherapy compared to chemotherapy or immune checkpoint blockade alone.

**Figure 1:**
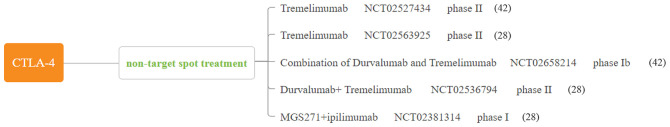
Major published clinical trials using CTLA-4 inhibitors in TNBC.

**Figure 2:**
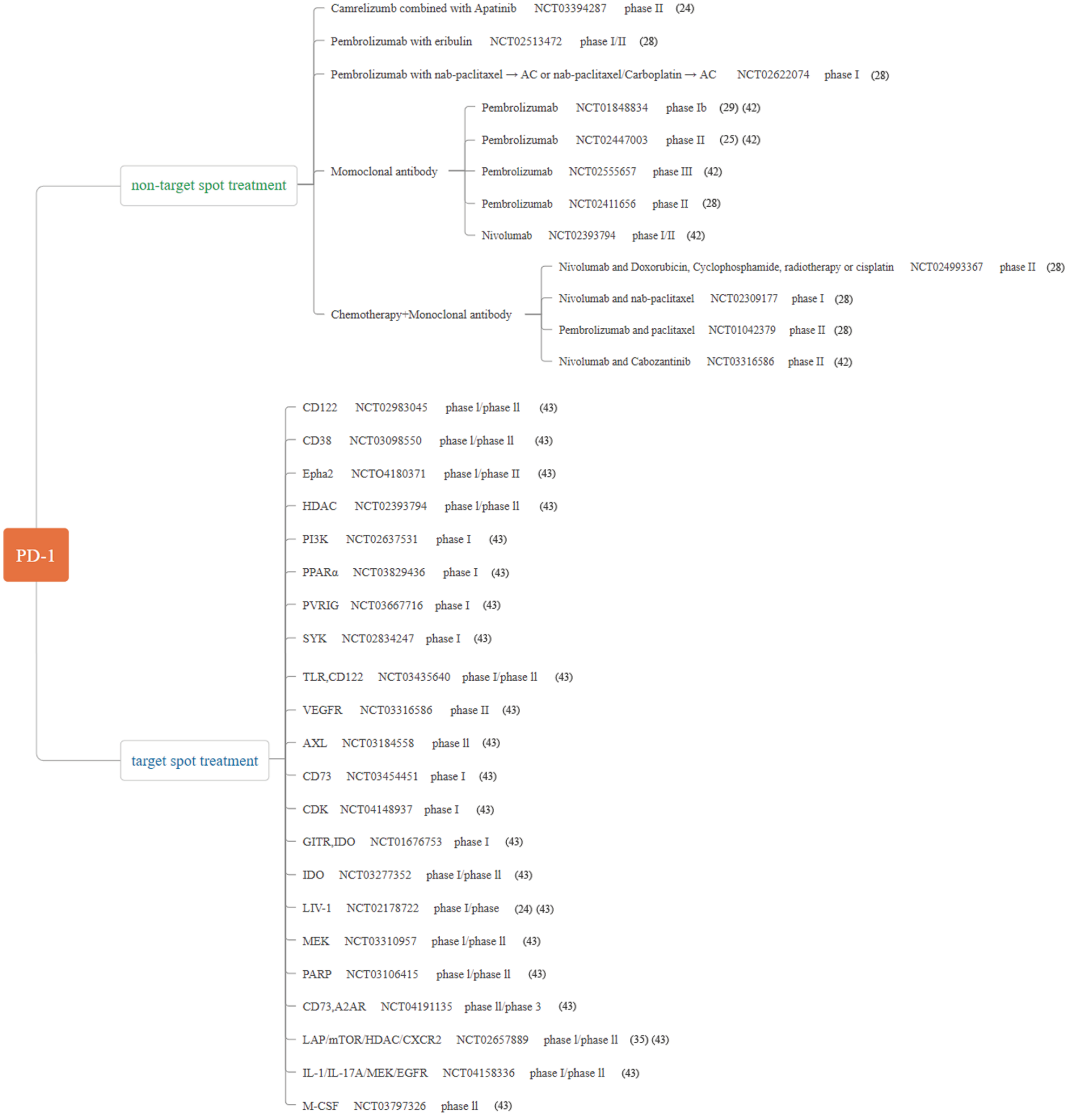
Major published clinical trials using PD-1 inhibitors in TNBC.

**Figure 3:**
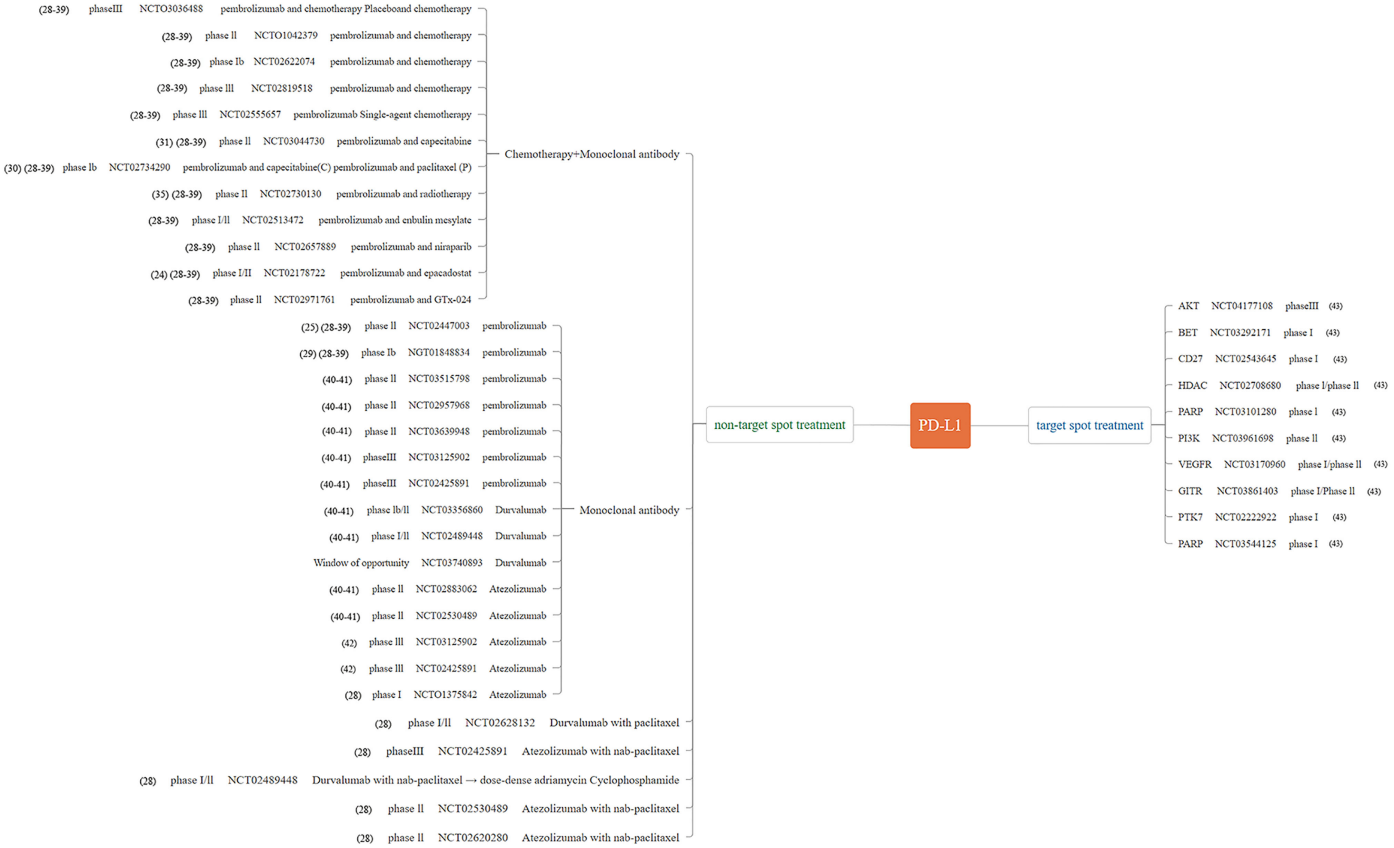
Major published clinical trials using PD-L1 inhibitors in TNBC.

We summarize the relevant clinical studies in the treatment of TNBC by ICIs in recent years (**Figures 1**–**3**) (28–43).

### Adoptive Cellular Immunotherapy (CD8^+^ T Cells)

The tumor immune microenvironment (TIME) plays a critical role in the progression, response to therapy and prognosis of most cancer patients. Tumor-infiltrating lymphocytes (TIL) are one of the predominant components of TIME, and the density and types of lymphocytes in the TIL fraction of a tumor have marked prognostic associations in breast cancer. This is especially actual of TNBC, which has the largest number of TILs. CD8 T cells are necessary immune cell in TIL (44). The infiltration of CD8^+^ T lymphocytes into solid tumors is related to the good prognosis of various types of cancers including TNBC (45). The T cell antigen receptor (TCR) of CD8^+^ T cells recognizes an antigenic peptide containing approximately 13–17 amino acids. It consists of major histocompatibility complex I molecules (MHC-I). Some research have observed that in TNBC transgenic mouse model, the levels of IFN-γ and TNF-α increased tumor-invasive CD8^+^ T cells, and subsequently led to apoptosis (46). Inhibition of Tregs (Regulatory cells) amplification can enhance the anti-tumor response of CD8^+^ T cells, thus affecting the growth of primary breast tumors or the metastasis of cancer cells to the lung (47, 48). Some studies have shown that LXR-inverse (Liver-X-Receptors) activation stimulates immune-mediated tumor destruction by means of improving CD8 T-cell activity in TNBC (49). It has currently been proved that PARP inhibitor olaparib induces CD8^+^ T cell infiltration in TNBC model with a BRCA1-deficient (breast cancer 1) deficiency by activating STING (interferon gene) pathway. Similarly, the efficacy of PARP inhibitors depends on the recruitment of CD8^+^ T cells in BRCA deficient TNBC model by activating intracavitary STING pathway (50, 51). STING-targeted immunotherapy enhances anti-tumor immunity mediated by natural killer cells and CD8^+^ T cells. It provides a theoretical basis for combining PARP inhibitors with CAR-T (Chimeric antigen receptor T) cell remedy to deal with TNBC disease.

### CAR-T and CAR-NK

CAR-T immunotherapy, which directly retargets the immune system od the patient to perceive and eradicate tumor cells with tumor-associated antigens (TAAs), and is presently being explored as a treatment for TNBCs (52, 53). However, immunotherapy is a new technology, and many bottlenecks remain to be overcome. For example, identifying specific goal tumor antigens and designing effective CAR is one of the many challenges of CAR-T therapy. Studies have shown that epidermal growth factor receptor-CAR (EGFR-CAR) lentivirus-infected T cells have a robust specific inhibitory effect on the growth of TNBC cells and tumor occurrence *in vitro* and *in vivo* (54). Some s find out about exhibit that ICAM1 (intercellular adhesion molecule-1)-specific CAR-T cells have been in a position to efficiently recognize ICAM1 expressing TNBC cells, and they can effectively minimize the growth of TNBC tumor inside and outside (55). Recently, the University of Pennsylvania completed a first phase scientific trial, which studied the security of injecting c-Met-CAR-T cell into TNBC patients [NCT01837602] (56). Results Inflammatory reaction was induced in TNBC tumor, and there was no evidence that drug-related side effects were greater than grade 1. Up to now, the research on MUC1 (Mucin1 glycoprotein)-CAR-T cell therapy has been the most investigated in clinical trials (57). The safety and efficacy of autologous MUC1-CAR-T cells are proposed to be evaluated in a phase I/II study in patients with relapsed or refractory TNBC [NCT02587689] (56). In addition, the inhibition of TGF-β-receptor signaling augments the anti-tumor function of ROR1 (receptor-tyrosine-kinase-like orphan receptor 1)-specific CAR T-cells against TNBC (58). Moreover, when recruiting participants, the safety and tolerance of allogeneic gamma delta (γδ) T cells transduced with CARs targeting NKG2D ligands on TNBC cells will be investigated in Phase I clinical trials [NCT04107142] (59, 60). The number of clinical trials of TNBC that CAR-T cell therapy is increasing, which may produce some exciting clinical effects. In addition, NK cells play a prominent role in the innate immune system because multiple receptors on the surface of the NK cells have been approved to kill cancer cells by interacting with their ligands of cancer cells, leading to apoptosis o cancer cells. Studies have shown that tissue factor as a new target for CAR-NK cell immunotherapy of TNBC (61). As EGFR is a potential therapeutic target for TNBC, EGFR-specific CAR NK cells (EGFR-CAR NK cells) is a promising strategy to inhibit tumor growth in breast cancer cell line-derived xenograft (CLDX) and patient-derived xenograft (PDX) mouse models (62).

## Combination Therapy

In targeted therapy of TNBC, there are some small molecule therapeutic targets, namely, PARP, DNA (cytosine-5)-methyltransferase 1(DNMT1), epidermal growth factor (EGF) and EGF receptor (EGFR), fibroblast growth factor receptor (FGFR), vascular endothelial growth factor (VEGF), and VEGF receptor (VEGFR) (63, 64). In TNBC subtype, basic helix-loop-helix (bHLH) transcription factors inhibitor of differentiation 1 (ID1) and inhibitor of differentiation 3 (ID3) (referred to as Id) play a vital role in maintaining cancer stem cell (CSC). Many molecules have been in preclinical trials. The application of ispinesib (a small molecule inhibitor in the ID1+ CSC results) to target the ID/Kif11 pathway, combined with chemotherapy, gave better response in TNBC subtype (65). This targeting ID1–Kif11 molecular pathway in the ID1+ CSCs, combined with chemotherapy and small molecular inhibitor, may reduce TNBC effect more effectively.

In addition, another promising strategy for combination therapy is to turn the “cold” tumors “hot” (66). Through a variety of methods, such as attracting T cell to the tumor through chemotherapy, radiation therapy, vaccines, and oncolytic viruses and bispecific antibodies (67). Other combination strategies include inhibition of other checkpoints or other immunosuppressive mechanisms, or enhancement of the activity of other checkpoint agonists, combination therapy to overcome T cell exhaustion, or conversion of immunosuppression [e.g., regulatory T cells (Tregs), myeloid-derived suppressor cells] into immunoreactive phenotypes (68, 69). In TNBC, however, chemotherapy combination of Atezolizumab enhanced the antitumor efficacy of Nab-paclitaxel only in patients with PD-L1 expression on tumor-infiltrating immune cells [NCT03371017] (26). On the other hand, chemotherapy combination of pembrolizumab paclitaxel protein-bound, or paclitaxel, or gemcitabine plus carboplatin also benefit patients with TNBC [NCT02819518] (70). Above all, tumors that respond to immune checkpoint inhibitors are typically so-called thermal or “hot” tumors with CD8 T cell infiltration, indicating that tumor cells are recognized by the immune system. CD8 positivity is often assessed as a predictor of response and a pharmacodynamic marker of response to combination therapies, which are hypothesized to enhance T cell infiltration and heat so-called “cold” tumors (71). Similarly, TNBC features immunological “cold” tumor, which with limited tumor infiltrating lymphocytes (72). To address this problem, we need to find a methodological strategy that actively recruits CD8^+^ T cells into the tumor microenvironment (TME), reverses “cold” tumors into “hot” tumors, and significantly improves their reactivity to ICIs (73).

## Potential Therapeutic Directions and Possible Strategies

### Mesothelin and TNBC

Tumor-associated antigen-mesothelin (MSLN) is a glycoprotein that exists on the cell surface and is highly expressed in various tumor tissues such as mesothelioma, non-small cell lung cancer, pancreatic cancer, and metastatic triple negative breast cancer, while no longer expressed in normal tissues or is low expressed in mesothelial tissues (74). Due to the characteristic of MSLN, it has become the focus of specific targeting antigen of tumor cells. Recent lookup used to be discovery of MSLN, a carcinogenic glycosyl-phosphatidyl-inositol (GPI) is overexpressed in TNBC (75). Above all, MSLN additionally play an vital position in T cell cloning and expansion and effector function, including initiating T cell activation (76). MSLN immune-targeted therapy (mAbs, CAR-T, vaccine) has top notch potential, and many of them have entered clinical trials of pancreatic cancer and lung cancer (76, 77). As a new personalized therapy, MSLN targeted therapy may achieve positive clinical results in TNBC patients.

### TNBC and Immune Viral Therapy

Recent trends in viral genetic engineering have allowed the development of oncolytic viruses with enhanced recognition capability to receptors overexpressed in tumor tissues, and viruses encoding or packaging suicide or pro-apoptotic genes or agents for delivery to cancer cells (78). Viruses can be manipulated to upregulate antigen presentation and T cell anti-tumor response. Talimogene laherparepvec (T-Vec, OncoVEXGM-CSF, Imlygic), an attenuated and genetically engineered herpes simplex virus (HSV) that overexpresses granulocyte-macrophage colony-stimulating factor (GM-CSF), is the only oncolytic virus approved for clinical use in the United States and Europe (ClinicalTrials.gov:NCT00769704) (79–81). Some studies have shown that cell vaccines primarily based on oncolytic vesicular stomatitis virus can improve the prognosis of TNBC by enhancing the functions of natural killer cells and CD8^+^ T cells (82). An oncolytic herpes simplex virus, which encodes the fundamental anti-tumor cytokine, interleukin 12 (IL-12), (designated G471-mIL12), can selectively kill cancer cells while inducing anti-tumor immunity (83), which is mainly manifested by the upregulation of CD8^+^ T cells activation markers in tumor microenvironment and the inhibition of tumor angiogenesis (84). Immunovirotherapy may be a promising method to treat TNBC patients.

### TNBC and Vaccines

Some studies have shown that mixed 19-peptide vaccine alone can achieve positive results in the treatment of refractory TNBC (85). The multi-epitope DNA and peptide vaccines is composed of the most immune dominant epitopes of SYCP1 (Synaptonemal Complex Protein 1) and ACRBP (Acrosin Binding Protein). As two conventional cancer/testis antigens, it can effectively activate the cellular and humoral immune response against 4T1 mouse breast tumor. In addition, this preventive combined immunization can drastically inhibit the growth of this mouse triple negative breast tumor (86). However, there are still some problems to be solved about vaccines, such as time, administration frequency and combination therapy strategy.

## Conclusions

**Figure 4:**
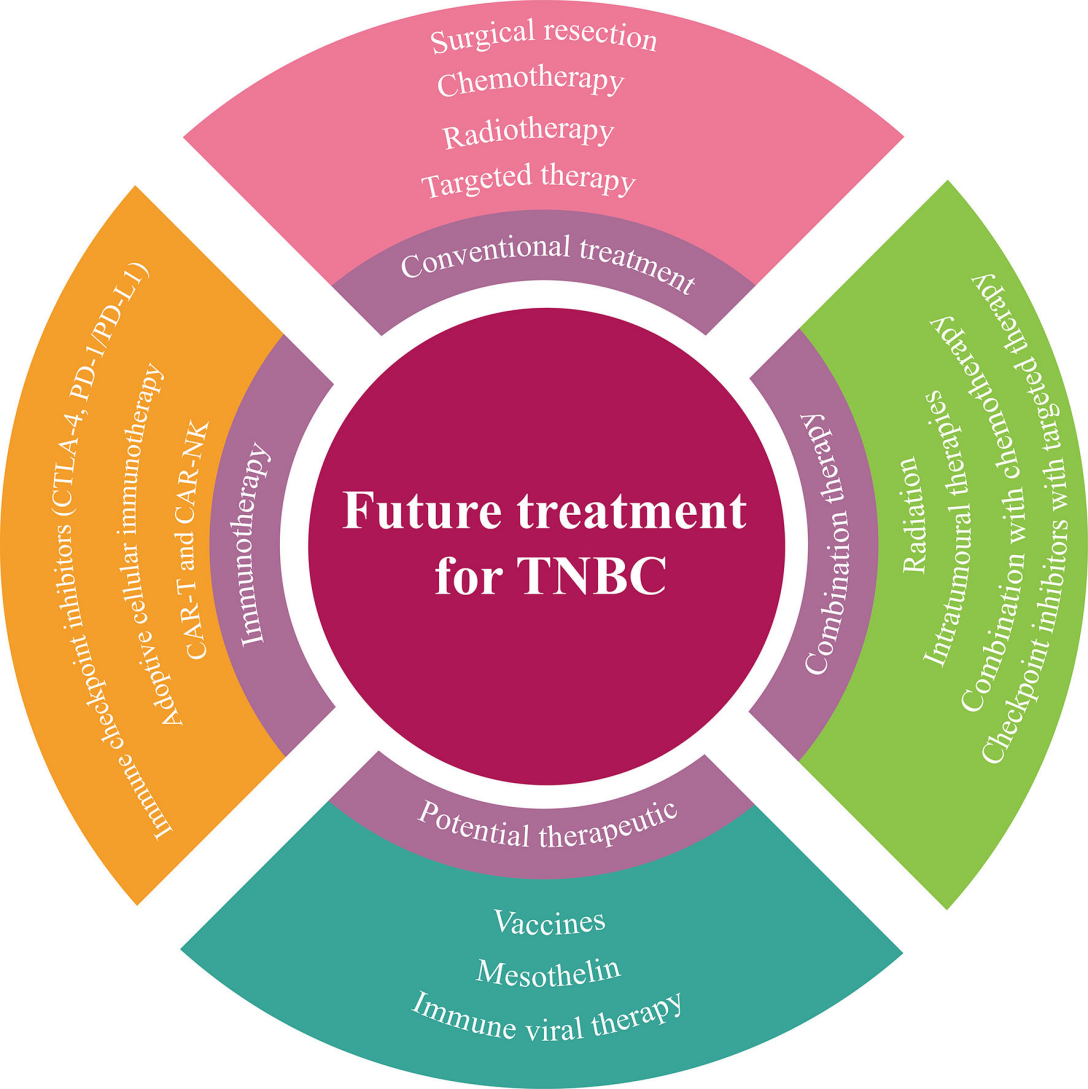
A summary of future treatment strategies for TNBC.

Recently, immunotherapy has delivered new hope to TNBC. The application of ICIs in TNBC will bring new light and advantage to patients. TNBC is currently exploring other new immunotherapy strategies, consisting of oncolytic virus and adoptive cell therapy, such as TIL metastasis and carcinoembryonic antigen T cells. Breast cancer vaccine constitutes another new therapeutic strategy to enhance anti-cancer immunity. Although the new preliminary immunotherapy still needs extensive clinical verification, these immunotherapies will promote the understanding of anti-cancer immunity of breast cancer and contribute to the development of effective strategies in the future. Further understanding of the mechanisms underlying immu-oncology are warranted to identify new immunotherapy-sensitive tumor types, combinations of different therapies will also become a promising strategy in the treatment of TNBC (**Figure 4**).

## Author Contributions

DQ: Writing - original draft, Writing—Review & editing. GZ, XY, and XX: Resources, Writing—Original draft. XM, SL, JW, XL, WW, JL, and YM: Participate in article writing. MM: Conceptualization, Writing—Original draft, Writing—Review & editing, Project administration, Funding acquisition. All authors contributed to the article and approved the submitted version.

## Funding

This work was supported by the National Natural Science Foundation of China (nos. 82074430, 81803979, 81673979, 82073748 and 81741130); the Natural Science Foundation of Guangdong Province, China (nos. 2018A030313393 and 2016A030313114); Science and Technology Program of Guangzhou, China (nos. 201803010051, 201707010245, and 201704020117) and the Fourth Batch of TCM Clinical Outstanding Talent Program of China (no. 444258); The Guangdong Basic and Applied Basic Research Foundation (no. 2019A1515011866 and 2021A1515010993); Guangdong Science and Technology Innovation Strategy Special fund (International science and technology cooperation projects, no. 2021A0505030034) Jinan University’s the National Collegiate Innovation and Startups Training Program (no. 202110559093). Thanks for the general Project of Natural Science Foundation of Guangdong Province, China in 2022.

## Conflict of Interest

The authors declare that the research was conducted in the absence of any commercial or financial relationships that could be construed as a potential conflict of interest.

## Publisher’s Note

All claims expressed in this article are solely those of the authors and do not necessarily represent those of their affiliated organizations, or those of the publisher, the editors and the reviewers. Any product that may be evaluated in this article, or claim that may be made by its manufacturer, is not guaranteed or endorsed by the publisher.
